# Shutter-Synchronized Molecular Beam Epitaxy for Wafer-Scale Homogeneous GaAs and Telecom Wavelength Quantum Emitter Growth

**DOI:** 10.3390/nano15030157

**Published:** 2025-01-21

**Authors:** Elias Kersting, Hans-Georg Babin, Nikolai Spitzer, Jun-Yong Yan, Feng Liu, Andreas D. Wieck, Arne Ludwig

**Affiliations:** 1 Lehrstuhl für Angewandte Festkörperphysik, Ruhr-Universität Bochum, Universitätsstraße 150, 44801 Bochum, Germany; elias.kersting@rub.de (E.K.); hans-georg.babin@rub.de (H.-G.B.); nikolai.spitzer@rub.de (N.S.); andreas.wieck@rub.de (A.D.W.); 2 State Key Laboratory of Extreme Photonics and Instrumentation, College of Information Science and Electronic Engineering, Zhejiang University, Hangzhou 310027, China; jun-yong@zju.edu.cn (J.-Y.Y.); feng_liu@zju.edu.cn (F.L.)

**Keywords:** molecular beam epitaxy, quantum dots, local droplet etching, telecom O-band, photoluminescence spectroscopy

## Abstract

Quantum dot (QD)-based single-photon emitter devices today are based on self-assembled random position nucleated QDs emitting at random wavelengths. Deterministic QD growth in position and emitter wavelength would be highly appreciated for industry-scale high-yield device manufacturing from wafers. Local droplet etching during molecular beam epitaxy is an all in situ method that allows excellent density control and predetermines the nucleation site of quantum dots. This method can produce strain-free GaAs QDs with excellent photonic and spin properties. Here, we focus on the emitter wavelength homogeneity. By wafer rotation-synchronized shutter opening time and adapted growth parameters, we grow QDs with a narrow peak emission wavelength homogeneity with no more than 1.2 nm shifts on a 45 mm diameter area and a narrow inhomogeneous ensemble broadening of only 2 nm at 4 K. The emission wavelength of these strain-free GaAs QDs is <800 nm, attractive for quantum optics experiments and quantum memory applications. We can use a similar random local droplet nucleation, nanohole drilling, and now, InAs infilling to produce QDs emitting in the telecommunication optical fiber transparency window around 1.3 µm, the so-called O-band. For this approach, we demonstrate good wavelength homogeneity and excellent density homogeneity beyond the possibilities of standard Stranski–Krastanov self-assembly. We discuss our methodology, structural and optical properties, and limitations set by our current setup capabilities.

## 1. Introduction

Photonic quantum technologies hold immense promise for advancing quantum communication, as described in Kimble’s vision of the quantum internet [1], as well as for applications in quantum sensing [2] and quantum computation [3]. At the core of these technologies is the requirement for reliable single-photon sources (SPSs) or entangled photon sources, both of which enable the creation and manipulation of quantum states for various applications.

Semiconductor quantum dots (QDs) [4], particularly those fabricated using metal organic chemical vapor deposition or molecular beam epitaxy (MBE), are excellent candidates for deterministic SPSs [5,6,7,8,9,10,11,12]. They can achieve high efficiency and deliver photons with exceptional quality [13,14,15]. The MBE growth method enables the production of high-quality heterostructures [16,17] with precise control over material fluxes, achieving sub-monolayer accuracy. Additionally, advanced charge control techniques allow for nearly transform-limited linewidths, which are critical for attaining high interference contrast in quantum optical applications [18,19,20]. This capability is particularly important in setups requiring multiplexing photons from different SPSs [20].

Moreover, quantum dots allow for electron spin control [21], enabling spin–photon entanglement and the realization of advanced photonic resource states [22,23]. Tailored protocols for QDs are now being developed to tolerate photon loss and even errors in quantum computation and communication [24]. These developments have the potential to address scalability challenges in photonic quantum technologies. Multiplexing photons from emitters with identical wavelengths can boost photon rates and support the generation of advanced quantum-entangled states, which are essential for scaling photonic quantum computation.

However, the growth of QDs faces challenges due to their random nucleation, which leads to variations in the size and wavelength of the emitter [25]. A variety of wavelength tuning approaches have been developed [26,27,28,29,30] with tuning of up to 25 meV [31], while charge control in QDs may require coupling to a reservoir limiting the tuning range to 4 meV [19]. To utilize QDs for, e.g., multiplexing, methods for tuning multiple emitters to identical wavelengths have been successfully demonstrated on chip [32,33], and significant advances in collective emission dynamics and dipole–dipole interactions have been reported [34]. The first commercial single-photon source products based on randomly nucleated QDs have emerged, with emission wavelengths typically in the range of 900–1000 nm. This range aligns with the availability of inexpensive Si-based single-photon detectors and the occurrence of QD transition energies below the GaAs bandgap. However, other applications, such as quantum information storage in Rb-atom clouds, require specific wavelengths near the Rb D1 (780 nm) and D2 (795 nm) transitions. QDs emitting in these spectral ranges have been realized by local droplet etching (LDE) strain-free GaAs QD growth [35]. For long-distance telecommunication, emissions in the transparency windows of optical fibers, such as the telecom O-band (~1310 nm), are ideal. InAs-QDs in GaAs-based semiconductors need some extra effort to reach this wavelength, such as a strain reduction layer (SRL) [36,37,38].

Efforts to predetermine QD nucleation sites have shown progress [39,40,41,42,43,44,45], but randomly nucleated QDs remain the best-performing emitters to date. The QD wafers for commercial SPS applications are typically produced using MBE, which allows for precise material deposition. However, unavoidable flux and temperature variations and drifts, as well as nonlinearities in strain-driven QD nucleation, present challenges [46,47]. These require stringent control over deposition parameters and temperature to achieve low-density QD growth over large surface areas, as shown by recent studies [48]. For SPS fabrication from randomly nucleated QDs, a density in the range from 0.1 to 10 QDs/µm^2^ is required. A density of 0.1 QD/µm^2^ is ideal for cathodoluminescence localization and deterministic fabrication [49,50], 1 QD/µm^2^ is ideal for optical isolating single QDs and deterministic fabrication [51,52], and ~10 QDs/µm^2^ is ideal for random QD waveguide fabrication [53].

For further scaling, robust methods to produce wafers with excellent homogeneity in both density and wavelength are needed. Local droplet etching (LDE) nanohole filling [35] offers a promising route to achieve this. This approach locally modifies the surface to control nucleation at later stages. Studies show that gradient deposition can lead to wavelength shifts for both strain-free and strained QDs grown in LDE nanoholes [54,55]. By fine-tuning these processes, homogeneous, low-density QD wafers with emission wavelengths suitable for Rb quantum memory and telecom applications can be fabricated.

## 2. Materials and Methods

### 2.1. Sample Growth

All samples presented herein were grown using a customized III/V Riber Epineat MBE system. The system contains effusion cells for the group III materials, specifically aluminum (two individual cells), gallium, and indium. The standard growth rates are 0.2 nm/s for GaAs, 0.1 nm/s for AlAs, 0.012 nm/s for InAs, and 0.3 nm/s for Al_33_Ga_67_As. In the context of n-type doping, silicon is employed. Arsenic is supplied by an arsenic valved-cracker cell, operating at a cracker temperature of 700 °C. At this temperature, the predominant species emitted is As_4_. A needle valve enables the precise adjustment of the arsenic flux. The utilized substrates were 3″ undoped (001)-oriented GaAs wafers with a thickness of 625 µm and a miscut of less than 0.1°. Prior to the growth process, the wafers were subject to a deoxidation procedure at a maximum temperature of 640 °C and an arsenic beam equivalent pressure (BEP) of approximately 1.0 × 10^−5^ torr. Temperature measurements were performed using a pyrometer for all GaAs quantum dot samples and band-edge infrared thermometry for the InAs quantum dot samples.

### 2.2. Material Deposition Methods

We distinguish between three different methods of material deposition during growth, which range from strong gradients in layer thickness and material composition to the most homogeneous layers possible in our setup.

**Figure 1:**
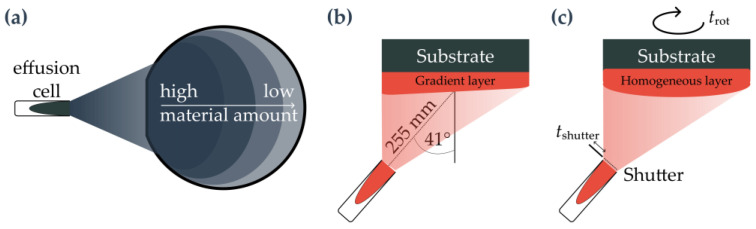
Schematic representation of material deposition: (**a**) Top view of the resulting material amount profile when the wafer is aligned in a fixed position to the effusion cell. (**b**) Side view of the material amount profile during gradient deposition and alignment of the cell to the substrate. (**c**) Side view of the material amount profile of a homogeneous layer grown under rotation. $t_{\mathrm{rot}}=t_{\mathrm{shutter}}$ applies to the shutter-synchronous deposition.

**Figure A1:**
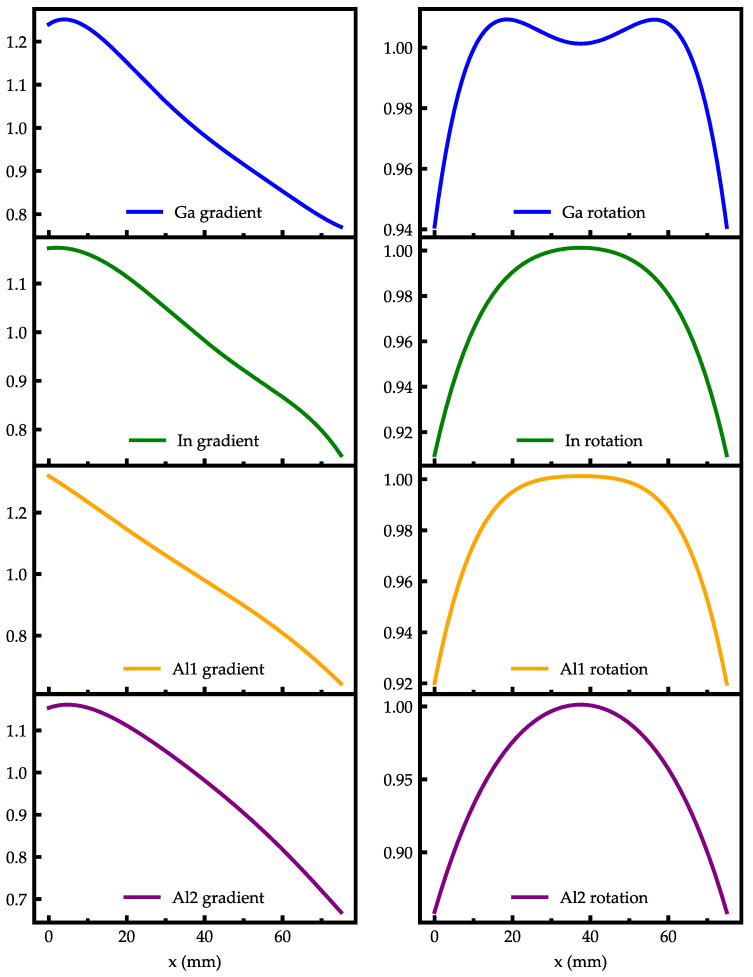
Normalized material amount profiles along the center of the wafer for gradient deposition (**left**) and shutter-synchronous deposition (**right**) for the different effusion cells. The profiles are calculated from quantum well PL measurements. The dip in the Ga cell flux distribution results from a too-hot center wafer temperature for the evaluated QW.

To achieve the maximum feasible gradients in layer thickness and composition, a rotation stop is used (see Figure 1a). The effusion cells are angled at 41° relative to the surface normal of the substrate with a distance of 255 mm between the cell opening and substrate center (see Figure 1b). Thus, thickness and concentration gradients are produced as a consequence of the wafer orientation to the cells and the angle between the individual cells. In conjunction with thickness simulations and calculations (see Figure A1), the gradient deposition method enables a fast and comprehensive parameter study (e.g., etch material amount and nanohole filling amount) and fine-tuning of the material amounts needed for the epitaxial layers. In this instance, the method is employed for the deposition of the layer situated directly below the quantum dots, the LDE etching material, and the filling of the etched nanoholes. The first described material gradient results in a sinusoidal modulation of the roughness, which is generated by alternating between completely and incompletely filled layers. As proposed by Bart et al. [56], this layer is called the pattern definition layer (PDL), which influences the QD nucleation and modulates the density and wavelength of the QDs [54,56]. A combination of gradient and deposition under rotation is possible as well, allowing for gradually adjusting the steepness of the layer gradients.

In the most common case, the wafer is rotated to obtain uniform layer thicknesses and to compensate for local parameter irregularities such as temperature (see Figure 1c). For this, the deposition of the material under rotation at a fixed speed is sufficient regardless of the duration of the grown layer, which results in a near-homogeneous thickness and material composition concentration. If deposition duration (shutter opening times) and rotation duration are not coordinated, small gradients will still form. These are frequently insignificant for conventional layers but can be significant for certain special layers, such as the etching of nanoholes or the growth of quantum dots.

In the case of these layers, the rotation time $t_{rot}$ must be aligned with the shutter opening time $t_{shutter}$, which means that $t_{rot}=t_{shutter}$. This process is referred to as shutter-synchronous deposition. This method allows for the realization of highly homogeneous material deposition. The deposited material thickness profile on a wafer is illustrated in Figure 1c and depends on the effusion cell profile. The gradient slope can be adjusted by covering a full rotation stop (i.e., a relative material flux increase of 50% across a full 3″ wafer), and less steep gradients by combining rotation and stopped deposition to homogeneous deposition by rotation-synchronized deposition time. Due to the cell geometry, we approximate a 2% material amount homogeneity in our setup for a 56 mm (48 mm) diameter region in the case of the Ga- (Al-1-) 60 ccm cell and a 44 mm (34 mm) diameter for the In- (Al-2-) 35 ccm cells, respectively. These values result from the analysis of the sample for cell flux characteristics (see Figure A1).

### 2.3. Characterization

Photoluminescence (PL) measurements were carried out using a custom-built setup that allows automatic recording of single spectra from each point of a full 3″ wafer. The sample is cooled to a temperature between 80 and 100 K by liquid nitrogen, and the entire cryostat is mounted on x- and y-translation stages for positional control and automatized wafer mapping. Excitation is achieved through a CW laser operating at 518 nm with a spot diameter of approximately 100 µm and power ranging from 1 to 20 mW. The collected light is divided by a beam splitter and recorded by two spectrometers: one equipped with a Si-CCD for the wavelength range of 340 to 900 nm, and another with an InGaAs line array for the range of 900 to 1714 nm. This allows a full spectrum to be recorded at any point on the wafer surface. The complete wafer is scanned with a step width of 0.5 mm. Evaluation of the recorded spectra allows analysis of peak heights, emission wavelength, and full width at half maximum (FWHM) along the entire wafer.

Atomic force microscopy (AFM) measurements of the surface holes and surface QDs were conducted in non-contact mode. Scans with dimensions of 3 × 3 µm^2^ or 5 × 5 µm^2^ were analyzed to evaluate the density.

## 3. Local Droplet-Etched GaAs QDs

### 3.1. GaAs QDs: Sample Structure and Gradient Design

**Figure 2:**
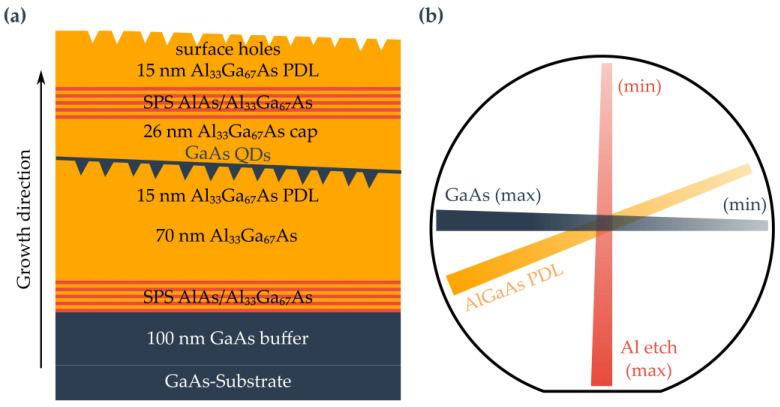
Growth and gradients: (**a**) Layer structure of GaAs QDs samples (not to scale). (**b**) Alignment of the material gradients.

The following sample structure, shown in Figure 2a, is used for the GaAs QDs. The growth was performed at a substrate temperature of 590 °C (pyrometer) and started with a 100 nm GaAs buffer layer to smoothen the re-growth interface of the substrate. After this, a short periodic superlattice (SPSL) with 20 layer pairs of 2.8 nm each AlAs and Al_33_Ga_67_As is followed. The SPSL forms a barrier for impurities from the substrate. The next layer is a 70 nm Al_33_Ga_67_As buffer layer followed by 15 nm (in the center) Al_33_Ga_67_As, which is deposited under a full stop gradient. This results in a PDL with a thickness difference of 6 nm and a roughness modulation period length of 3 mm. For the etching process, the substrate temperature is lowered to 545 °C pyrometer temperature, and the As-valve is partially closed to reduce the BEP to (4.5–4.8) × 10^−7^ torr. As it is not possible to measure the BEP during growth, it is determined after growth under similar conditions. For etching, 0.29 nm (equivalent to 1.025 ML, where ML stands for monolayer) Al is deposited in a gradient. Following the etching process, the As-valve opening returns to its previous value and the growth chamber pressure quickly returns to its original value. Next, the nanoholes are filled with GaAs in a gradient, resulting in a QD emission wavelength shift across the wafer. Three samples were grown with different filling amounts in relation to the center of the wafer: sample $\#A_{1.00/GaAs/grad}^{0.3/Al2/grad}$ with 1 nm, sample $\#B_{0.60/GaAs/grad}^{0.3/Al2/grad}$ with 0.6 nm, and sample $\#C_{0.44/GaAs/grad}^{0.3/Al2/grad}$ with 0.44 nm GaAs material amount. The superscript indicates the layer thickness equivalent etching quantity in nm, the etching material, and the deposition method. The subscript contains the fill amount, the filling material, and also the deposition method for filling nanoholes. “grad” here refers to gradient deposition, and “rot” refers to shutter-synchronous deposition. An overview of all sample parameters is presented in Table 1. The gradients and their directions are shown in Figure 1b. After the filling, the QDs are capped by a 26 nm Al_33_Ga_67_As layer. To estimate the hole dimensions via atomic force microscopy (AFM), an SPSL followed by a 15 nm PDL layer with Al-etched surface nanoholes were grown under identical conditions to the buried nanoholes.

Towards the main objective of this work, namely achieving homogeneous emission wavelength, a series of three samples with a similar structure but rotation-synchronized material deposition was produced. For the first sample, $\#D_{0.60/GaAs/rot}^{0.3/Al2/rot}$, 0.3 nm etch material was used. For samples $\#E_{0.60/GaAs/rot}^{0.4/Al2/rot}$ and $\#F_{0.60/GaAs/rot}^{0.4/Al1/rot}$, 0.4 nm material was used. The etch material of the last sample comes from aluminum cell 2. An overview of the growth parameters can be found in Table 1.

### 3.2. Influence of GaAs Fill Amount on the Emission Wavelength

**Figure 3:**
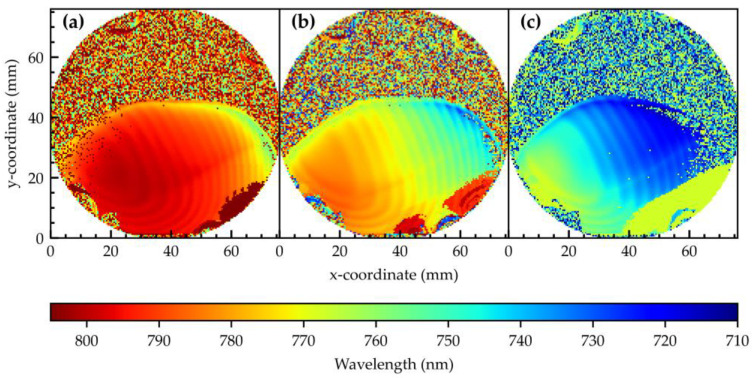
Wafer maps showing the s-peak photoluminescence emission wavelength distribution at ~100 K. In all samples, the filling material was deposited as a gradient with the following amounts in the center of the samples: (**a**) 1 nm GaAs; (**b**) 0.60 nm GaAs; (**c**) 0.44 nm GaAs.

Figure 3a–c shows the PL ground state emission wavelength of samples $\#A_{1.00/GaAs/grad}^{0.3/Al2/grad}$, $\#B_{0.60/GaAs/grad}^{0.3/Al2/grad}$, and $\#C_{0.44/GaAs/grad}^{0.3/Al2/grad}$ with a shared color bar. According to Figure 2b, the amount of aluminum etching along the wafer increases from top to bottom towards the orientation flat, also called the big flat. Above a certain etch material quantity, a threshold is exceeded, and nanoholes can be drilled. This is why the upper area of the wafer shows no QD-related emission. A transition from an area with QDs to an area with no QDs is visible for all samples at around y~45 nm. In this case, the critical Al-etch material amount is $\theta_{crit}\approx 0.29\;nm$. The quantity also depends on the As-flux during etching, as this also determines the effective etching amount. A detailed study including AFM images for surface morphology characterization and density determination of sample $\#C_{0.44/GaAs/grad}^{0.3/Al2/grad}$ (#15389) is presented in the work of Kruck et al. [57]. Due to the PDL, a wavelength modulation in the gradient direction with a ~3 nm amplitude on a 3 mm period can be distinguished. It also influences the PL intensity, which is discussed in more detail in the work of Babin et al. [54]. The artifacts in the lower right area of the wafer are most likely caused by an incompletely closed shutter during the PDL or QD growth and are of no further interest.

The key effect is the ground state emission wavelength shift following the GaAs filling gradient. Lower filling quantities result in higher emission energies. Approximately 1.20 nm material is deposited in the small flat area of the wafer $\#A_{1.00/GaAs/grad}^{0.3/Al2/grad}$, resulting in an emission wavelength of 805 nm. For sample $\#C_{0.44/GaAs/grad}^{0.3/Al2/grad}$, at a minimum filling amount of 0.4 nm, a minimum wavelength of 710 nm is obtained. This means a difference of 0.8 nm in the filling quantity leads to a wavelength shift of 95 nm. Sample $\#A_{1.00/GaAs/grad}^{0.3/Al2/grad}$ exhibits a 35 nm wavelength shift, while sample $\#C_{0.44/GaAs/grad}^{0.3/Al2/grad}$, with the lowest GaAs filling, shows the largest wavelength shift of 55 nm across the entire wafer. Sample $\#A_{1.00/GaAs/grad}^{0.3/Al2/grad}$ also displays a nearly homogeneous region in emission wavelength at the longest wavelength region, suggesting that the holes are overfilled in that region and the wavelength no longer shifts with the filling amount. Additionally, we observe deviations in the wavelength shifts from the GaAs gradient direction. This is due to the influence of the Al-etching material amount on the wavelength, although this impact is smaller [54,57]. These results are summarized in Table 2.

### 3.3. Shutter-Synchronized GaAs QDs

In Section 3.2, we demonstrate that the GaAs fill amount has a strong impact on the emission wavelength. Now, a shutter-synchronized deposition of the buffer layer (previously PDL), the Al-etch material, and the GaAs fill material are applied to homogenize the emission wavelength. A parameter study culminated in sample $\#F_{0.60/GaAs/rot}^{0.4/Al1/rot}$, with 0.4 nm Al-etch material deposited by the Al-1 cell and a 0.6 nm GaAs fill amount.

**Figure 4:**
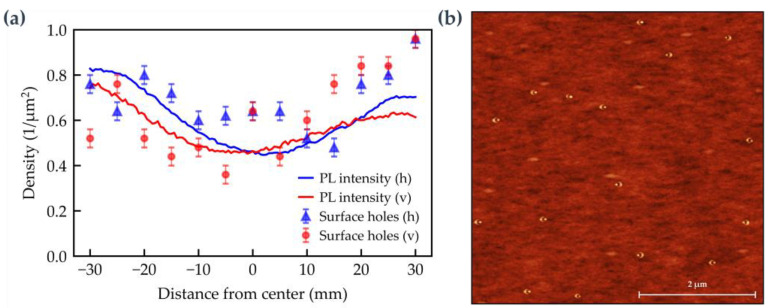
AFM and PL study of sample $\#E_{0.60/GaAs/rot}^{0.4/Al2/rot}$ to determine the QD density: (**a**) Surface hole density and integrated QD PL intensity in horizontal (h) and vertical (v) directions. (**b**) Representative 5 × 5 µm^2^ image of the surface morphology at center position (38|38). The color scale ranges from 0 nm (dark) to 8 nm (bright).

An AFM study is shown in Figure 4. Since the growth parameters of the etched holes and the surface holes are almost identical, AFM analysis of the surface can be used to approximate the QD density along the wafer (see Figure 4a). In the investigated range of ±30 mm from the center, the density varies only between 0.4 QD/µm^2^ and 1 QD/µm^2^. Furthermore, there is a consistency between PL intensity and QD density. A representative AFM measurement from the center is shown in Figure 4b.

**Figure 5:**
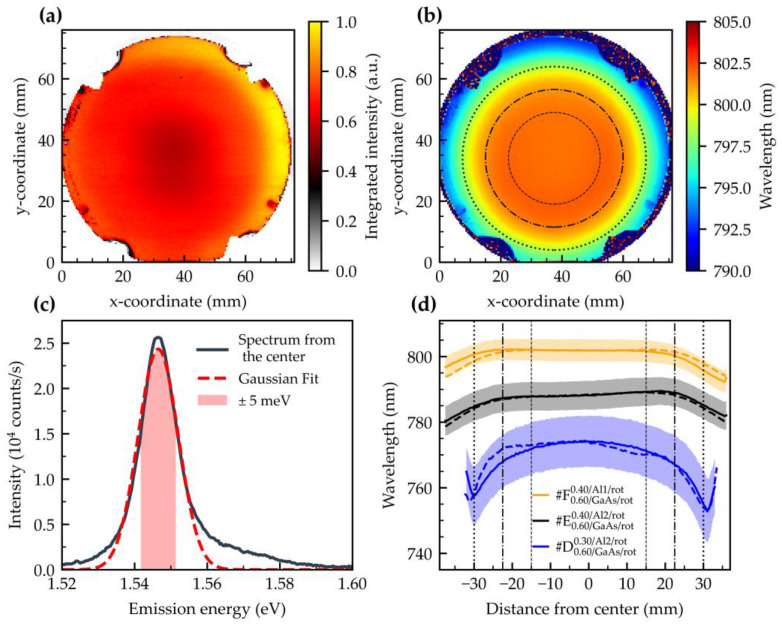
Photoluminescence results at ~90 K of the shutter-synchronized grown GaAs QDs: (**a**) Integrated intensity in a wavelength range of (750–820) nm of sample #F. (**b**) Wafer map of the s-peak emission wavelength of sample #F. The areas under investigation are marked by circles with diameters of 30, 45, and 60 mm. (**c**) Representative spectrum and Gaussian fitting from the center of the wafer (sample #F). The red shaded area corresponds to the investigated energy range (here, for example, a total tuning range of ±5 meV). (**d**) Emission wavelength profile in vertical (dashed) and horizontal (solid line) directions through the center of the wafer for samples #D, #E, and #F. The shaded areas correspond to the FWHM area of the ensemble PL.

With the exception of the substrate holder, the integrated PL intensity of this sample shows homogeneous QD emission on the entire wafer (Figure 5a). A small intensity drop in the middle of the wafer and an enhanced intensity of the upper and right edges of the wafer are attributed to inhomogeneities of the substrate temperature. According to the work of Kerbst et al. [58], a higher temperature leads to reduced nanohole density and therefore to lower QD density. This finally leads to lower PL intensity.

Figure 5b displays the Gauss-fit ground state emission wavelength. For our analysis of useful heterostructure material, we focus on the central region and areas with different-sized circles (diameters *D* = 30 mm, 45 mm, and 60 mm). For the central region, a peak wavelength of 801.7 nm and an inhomogeneous broadening of ~13 meV FWHM are found. For *D* = 30 mm, a mean wavelength of 801.84 ± 0.10 nm is calculated. This depicts an exceptionally uniform region, exhibiting an absolute peak wavelength variation of only about 0.6 nm. Increasing the area under consideration to 45 mm results in 801.79 ± 0.19 nm for the mean wavelength and absolute wavelength variation of 1.2 nm. Even for the largest area of 60 mm, there is an exceptional homogeneity with 801.1 ± 1.0 nm for the mean wavelength and 4 nm for the absolute wavelength variation. It is important to note that the FWHM does not vary significantly across the entire wafer area. All values can also be found in Appendix A in Table A1. Subsequently, a more pronounced wavelength shift is observed, which can be attributed to the radiation profile of the Ga and Al cells and the temperature decline towards the edge of the wafer.

Figure 5d illustrates the wavelength distribution for samples $\#D_{0.60/GaAs/rot}^{0.3/Al2/rot}$, $\#E_{0.60/GaAs/rot}^{0.4/Al2/rot}$, and $\#F_{0.60/GaAs/rot}^{0.4/Al1/rot}$. Sample #D shows a relatively homogeneous region of 45 mm diameter, and a steep wavelength drop afterwards. We relate this to an insufficient amount of Al-etch material in this area due to the sharp Al-2 cell profile (c.f. Figure A1). As a consequence, a transition is formed that, as is typical, is characterized by a blueshift and a decrease in intensity. With an Al-etch amount of 0.4 nm (samples #E and #F), no transition can be observed. To improve the homogeneity even further, an Al-1 cell with a more homogeneous profile was used for sample $\#F_{0.60/GaAs/rot}^{0.4/Al1/rot}$. In addition, the As-flux was lower in this sample, which led to an increase in the effective amount of etching material. This leads to the formation of deeper holes and a corresponding redshift of the emission wavelength between the samples $\#E_{0.60/GaAs/rot}^{0.4/Al2/rot}$ and $\#F_{0.60/GaAs/rot}^{0.4/Al1/rot}$, despite the same deposited Al-etch and Ga fill amounts. As the Al-etch material amount is sufficient for the whole wafer area, the blueshift towards the edges is mainly due to the inhomogeneous effusion profile of the Ga cell, with only minor contributions from the Al cells and temperature inhomogeneity.

The GaAs LDE QD density can be comfortably adjusted to values needed for single QD experiments or SPS fabrication either by temperature [59] or Al-deposition amount [57], and is not considered a particularly difficult parameter to control. We use QD PL intensity as a measure for QD density and therefore assume a strong indication for a homogeneous density for samples $\#E_{0.60/GaAs/rot}^{0.4/Al2/rot}$ and $\#F_{0.60/GaAs/rot}^{0.4/Al1/rot}$.

The QD density is thus not the limiting factor for SPS device fabrication from LDE QDs. The remaining quantity is the random wavelength distribution. QDs can be tuned by 25 meV [31], and on-chip electrical tuning of different emitters has been demonstrated [33]. However, for charge-stabilized QDs, much smaller tuning ranges are anticipated.

We calculate which portion of QDs on a wafer can be tuned into resonance considering different tuning ranges. We take the wafer center peak emission energy $E_{center}$ (as the design wavelength) and apply a tuning range window $E_{r}$ of different magnitudes to the Gaussian fits of the measured data. The proportion of QDs at a specific point is calculated as follows:

(1) $$P_{QDs}=\frac{\int_{a}^{b}A\cdot e^{-{\left(\frac{E-E_{0}}{c}\right)}^{2}}dE}{\int_{-\infty }^{+\infty }A\cdot e^{-{\left(\frac{E-E_{0}}{c}\right)}^{2}}dE}=-\frac{1}{2}\;\left[erf\left(\frac{E_{0-b}}{c}\right)-erf\left(\frac{E_{0-a}}{c}\right)\right].$$

**Figure 6:**
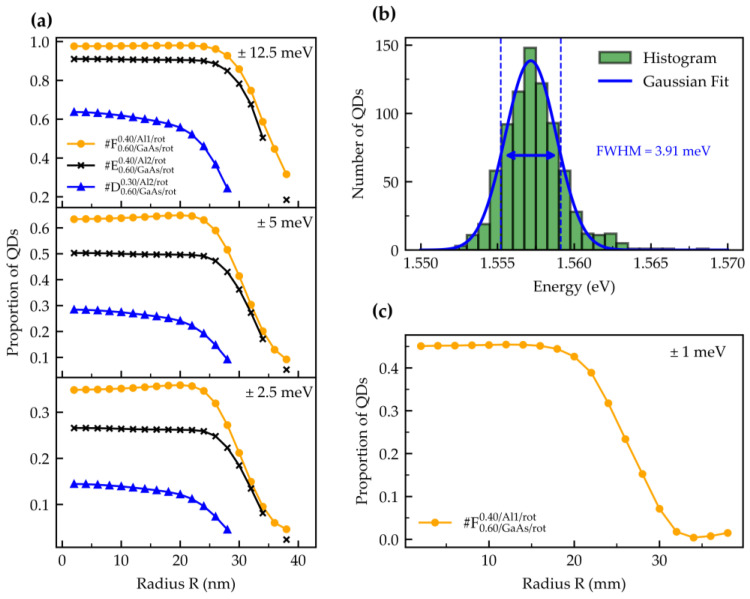
The proportion of QDs emitting in the range accessible by Stark-shift tuning for wafers #D, #E, and #F: (**a**) Proportion of QDs that are tunable to the wafer center peak wavelength, calculated from the PL measured at 90 K. A maximum tuning range of 25 meV has been reported. We assume a statistical distribution of the tuning range and calculate for a ±12.5 meV and smaller tuning ranges. (**b**) The PL ground state transition energy of a total of 794 individual QDs at position (38/42) near the center of wafer #F at a sample temperature of 4 K. The excitation was performed with a 637 nm laser operated at a power of 7 µW. (**c**) Proportion of QDs that can be tuned to the wafer center peak emission wavelength for a ±1 meV tuning range.

The integration limits are ${a=E}_{center}-E_{r}/2$ and $b=E_{center}+E_{r}/2$, and $E_{0},\;A$ and *c* are the parameters of the Gaussian function determined from the local fits. In Figure 6a, we plot the proportions of QDs that fall into the according windows. We focus on concentric ring sections, each 2 mm wide, where the region at *R* = 2 mm spans from the center out to 2 mm, *R* = 4 mm spans from 2 to 4 mm, and so on. The aim is to determine up to which radius useful material is present. For a tuning range of ±12.5 meV, we find that nearly all QDs (98%) can be tuned into mutual resonance within a 50 mm diameter area.

The true energy transitions of QDs are obscured at temperatures above ~10 K due to random thermal fluctuations of charge carriers in and around the QDs. Our full 3" PL wafer mapper, limited to temperatures around 90 K, thus cannot assess the true QD transition energy inhomogeneity. The observed peak broadening at 90 K stems from multiple factors: random thermal carrier and charge distribution in the QD shells and around the QDs, high excitation power-related carrier fluctuations, and spectrometer resolution limitations. These factors collectively contribute to the broadening of the measured PL peaks, masking the intrinsic QD transition energies. Therefore, an automated PL measurement of 794 randomly selected individual QDs was performed at low temperature and excitation power ($\lambda_{\mathrm{excitation}}=637\;\mathrm{nm}$, *P* = 7 µW, and *T* = 4 K) at position (38, 42) of sample #F, and a corresponding histogram is shown in Figure 6b [60]. We can now state that our measured FWHM at that location corresponds to a spectral dispersion of only 3.9 meV (2 nm). As sample $\#F_{0.60/GaAs/rot}^{0.4/Al1/rot}$ is very homogeneous, we used the found FWHM ratio for 90 K and 4 K at this position to calculate the true QD emission energy/wavelength distribution on the wafer and plot the true tuning range-related useful QD proportion in Figure 6c. Under the assumption made, even for a small tuning range of only ±1 meV, at least 40% of all QDs at every point of the wafer within a diameter of 40 mm can be tuned into exact mutual resonance. At a typical area density of 1 QD/µm^2^, this would correspond to more than half a billion QDs.

## 4. Local Droplet-Etched InAs QDs

### 4.1. InAs QDs: Sample Structure and Gradient Design

**Figure 7:**
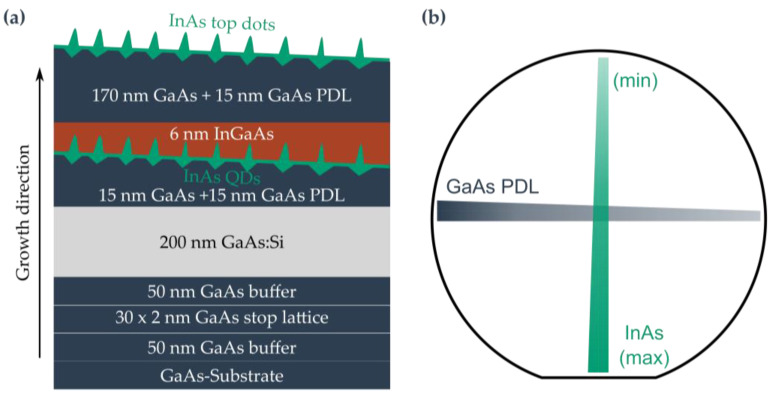
Growth and gradients: (**a**) Layer structure of the LDE InAs QDs (not to scale). (**b**) Alignment of the PDL and the InAs filling gradient.

In this section, we present two LDE InAs O-band QD wafer projects: one gradient and one shutter-synchronized rotation wafer. The sample structures of the LDE InAs QDs are similar to the structures of the LDE GaAs QD samples. Since the LDE InAs QDs were grown during a time when both of our Al cells were inoperable, the structure does not contain any Al. The gradient sample structure is exemplarily shown in Figure 7a. In detail, after oxide removal at 640 °C, a 50 nm GaAs buffer and a stop lattice of 30 times 2 nm GaAs were grown instead of the usual SPSL. The next layer is 50 nm GaAs followed by a 200 nm Si-doped GaAs layer. A barrier of 15 nm GaAs plus 15 nm gradient GaAs PDL was grown on top of this. The LDE process is similar to that described in Section 3.1: At an As-flux of 1.7 × 10^−7^ torr and a substrate temperature of 530 °C (band-edge thermometry measurement), 0.84 nm (3 ML) Ga was deposited under rotation. After 180 s, nanoholes were etched and subsequently filled with InAs in 12.25 cycles by a 4 s deposition and 4 s break each with a full stop gradient method (Figure 7b) corresponding to ~1.94 ML InAs at the center. This was performed at an As BEP of 6.8 × 10^−6^ torr and a measured substrate temperature of 495–500 °C. The QDs were overgrown with a 6 nm In_30_Ga_70_As strain reduction layer (SRL) followed by 170 nm GaAs. The PDL, LDE, and QD growth processes were repeated to obtain surface QDs for AFM analysis. Another sample with the same parameters was grown with shutter-synchronized rotation growth for the LDE and QD layer. The PDL was retained in this case. An overview of the growth parameters can be found in Table 3.

### 4.2. Influence of InAs Fill Amount on the Emission Wavelength

**Figure 8:**
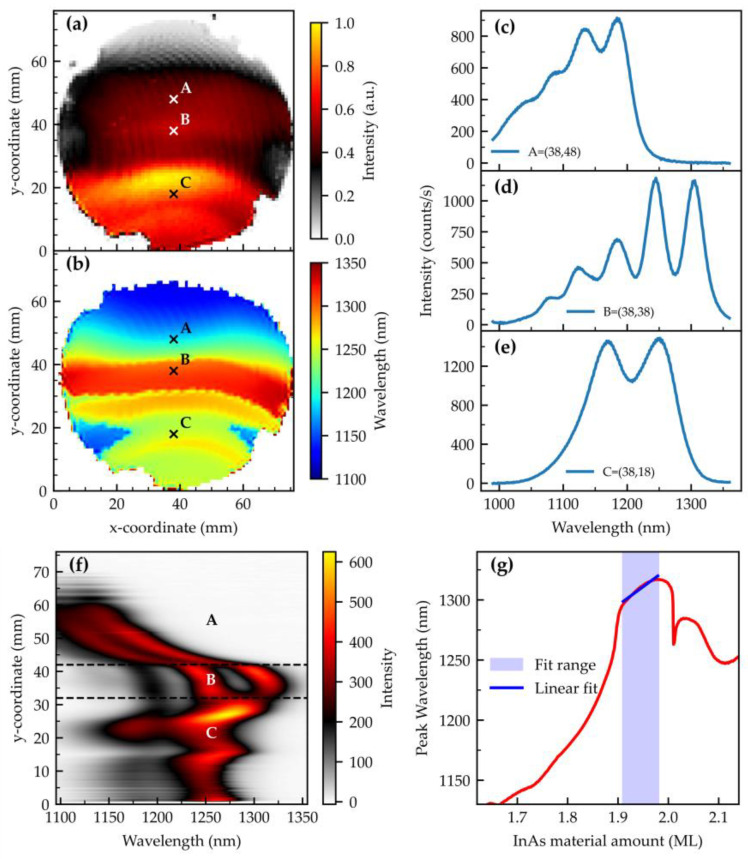
Photoluminescence results at ~80 K of the gradient-grown InAs QDs: (**a**) Integrated intensity in a wavelength range of (990–1360) nm. A, B, and C mark the 3 different areas. (**b**) Wafer map of the s-peak emission wavelength. (**c**–**e**) One representative spectrum each from areas A, B, and C. (**f**) Spectral heatmap in the vertical direction for x = 38 mm. (**g**) Wavelength at the maximum intensity over the InAs material amount.

**Figure A2:**
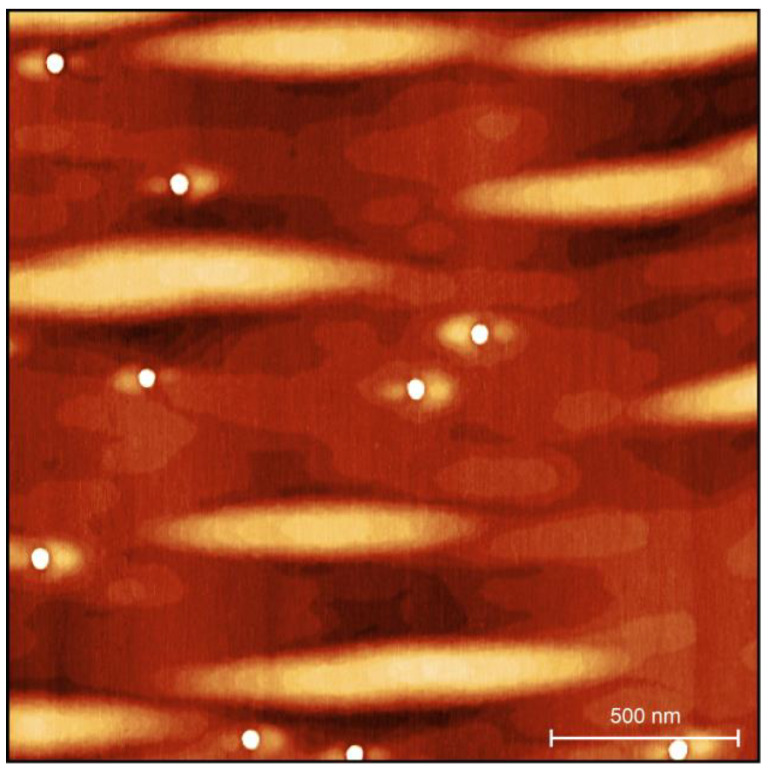
Surface morphology of sample $\#G_{12.25/InAs,grad}^{0.84/Ga/rot}$ (#15736) from area B. The buried QDs (elongated mounds) have a height of ~4 nm and the LDE QDs of ~15 nm. The color scale ranges from 0.5 nm (dark) to 5 nm (bright).

In this section, we revisit some recent findings on droplet-etched InAs O-band QDs already published by Spitzer et al. [55]. Sample $\#G_{12.25/InAs,grad}^{0.84/Ga/rot}$ shows three characteristically different areas which are marked by A, B, and C (see Figure 8a,b). The deposited InAs amount increases in the vertical direction from the top to the bottom (see Figure 7b). The characterization of the surface morphology of the different areas has already been published in the work of Spitzer et al. [55]. For the sample presented here, a representative AFM scan from area B is shown in Figure A2.

In area A, the amount of InAs is not yet sufficient to form standard Stranski–Krastanov (SK) InAs QDs. However, optically active QDs form in the previously etched holes, determining nucleation site and density. The observable s-peak emission wavelength follows the InAs gradient and ranges from 1100 to 1250 nm. This can be clearly seen in the vertical line profile of the PL emission in Figure 8f. A representative spectrum is shown in Figure 8c. The integrated PL intensity is weak and increases slightly towards area B (see Figure 8a). The transition from A to B is characterized by a strong wavelength gradient. After that, an approx. 10 mm wide strip is formed with peak emission wavelengths in the range from 1295 to 1315 nm, covering the desired range for O-band QDs. A representative spectrum is shown in Figure 8d. Up to five shell-related peaks can be clearly resolved. The overall intensity is slightly larger than for area A. The deposited amount of InAs is still not sufficient for the formation of standard SK QDs. This threshold is exceeded at the transition from area B to C. Initially, in that region an intensity drop is observed. The QDs in the filled holes are now too large and, according to our interpretation, no longer optically active. In the transition to region C, a blueshift and strong increase in intensity is visible. Here, standard SK QDs randomly nucleate at a high density, explaining the increased PL intensity. The nucleation also tends to occur around the mounds surrounding the nanoholes [55]. A clearly changed spectrum with a ground state peak emission wavelength in the range of 1250–1290 nm and two pronounced peaks with a larger separation than for the LDE-related PL is visible.

Regardless of the area, the PL shows periodic intensity modulations that can be attributed to the PDL. This is known for SK QDs [56] and LDE GaAs QDs [54]. Here, we observe for the first time that Ga-etched LDE InAs QDs are also modulated in intensity and wavelength.

As with the LDE GaAs QDs, a notable correlation between the emission wavelength and the InAs fill amount can be observed. For a more detailed examination, a line scan was carried out in the y-direction along the InAs deposition gradient with a step resolution of 0.1 mm. The profile plot is shown in Figure 8f. To determine the slope of the wavelength shift, the peak emission wavelength was plotted against the calculated amount of InAs at the corresponding y-positions (see Figure 8g). In the area of interest (B), a relatively small, linearly approximated slope of ~3 nm/mm on the wafer or, as calculated from the flux distribution, (308 ± 8) nm/ML is found. This corresponds to a well-tolerable wavelength uncertainty of ~1.3 nm per % flux uncertainty. While Figure 8g signifies the pronounced wavelength shift at the transitions from A to B and from B to C, to achieve homogeneous O-band emission on the wafer, the growth parameters must be selected so that one ends up in area B.

### 4.3. Shutter-Synchronized LDE InAs QDs

**Figure 9:**
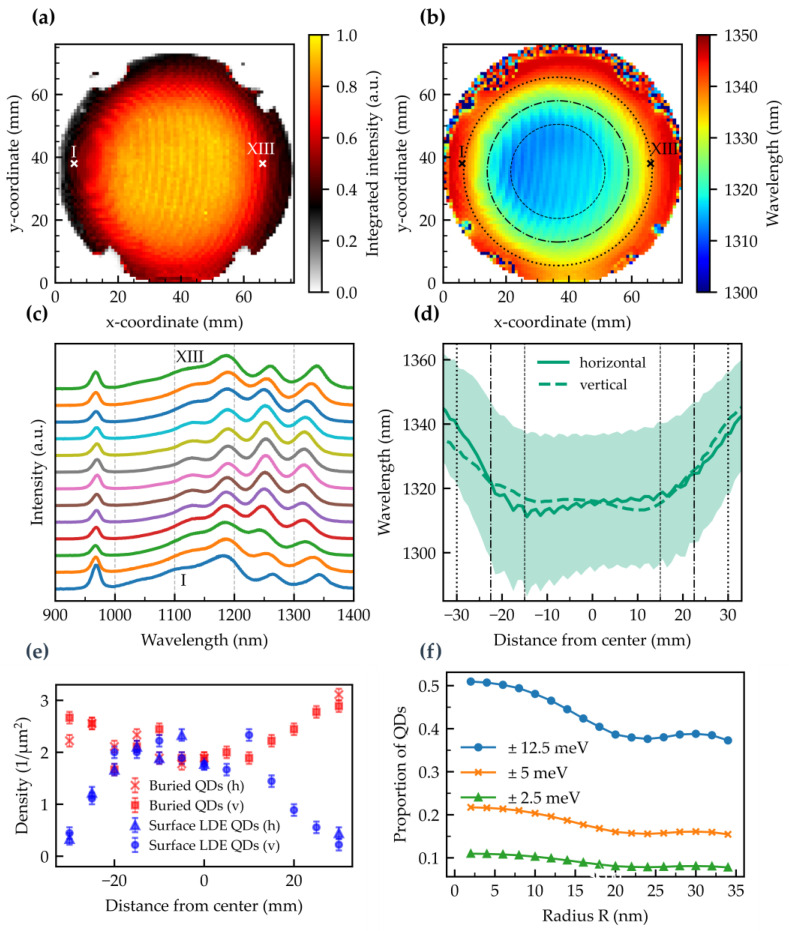
Photoluminescence results at ~80 K of the shutter-synchronized grown InAs QDs: (**a**) Integrated intensity in a wavelength range of (1100–1350) nm. For the offset plots in (**c**), orientation points are marked by I and XIII. (**b**) Wafer map of the s-peak emission wavelength. The areas under investigation are marked by circles with diameters of 30, 45, and 60 mm. (**c**) Offset spectra plots along the horizontal line from I to XIII with an increment of 5 mm. (**d**) Horizontal and vertical line profiles of the emission wavelength and FWHM (shaded area) through the center of the wafer. (**e**) Density of buried QDs and surface LDE QDs in horizontal (h) and vertical (v) directions through the center. (**f**) Proportion of QDs that are tunable to the wafer center peak wavelength for three different tuning ranges.

Figure 9a presents the integrated PL intensity of sample $\#H_{12.25/InAs/rot}^{0.84/Ga/rot}$. Besides the PDL-related modulation, the profile has a circular symmetry and excellent homogeneity in the central region. The intensity is higher at the wafer’s center, contrasting with the trend observed in Al-etched LDE GaAs QDs. Also, for the Ga-etched LDE QDs, a slightly lower density is expected in the center due to the higher temperature there. This disparity is likely attributable to another temperature effect, where a higher central temperature results in increased indium desorption and/or alloy intermixing [61], while the cooler wafer edges retain more InAs. This contrasts the sharp InAs profile of our setup with lower deposited material towards the edges.

In Figure 9b, the ground state emission wavelength also shows modulation from the PDL, correlating with the intensity modulation: lower PL intensity corresponds to a longer emission wavelength.

Figure 9c shows single spectra starting from point I = (6|38) to point XIII = (66|38) with a step size of 5 mm (see Figure 9b). Spectra with clearly resolved peaks can be seen over the entire range. A redshift of the ground state transition for the first and last two spectra is visible along with a larger level splitting. We attribute this effect to a lower temperature at the wafer edge. For the homogeneity analysis, the area within different-sized circles (diameter *D* = 30 mm, 45 mm, and 60 mm) is investigated (see rings in Figure 9b). We find a ground state peak wavelength of 1315 nm, and mean wavelengths of 1315.3 ± 1.6 nm, 1317 ± 3 nm, and 1323 ± 7 nm for the center and diameters of 30 mm, 45 mm, and 60 mm, respectively. The total peak wavelength variations are 8 nm, 14 nm, and 29 nm, respectively.

Figure 9d shows the horizontal and vertical wavelength profiles. The horizontal wavelength profile follows the PDL modulation, while the vertical profile is as well affected by the non-perfect perpendicular PDL, distorting the vertical profile slightly. A notable redshift towards the edges surprises, as the indium cell profile clearly decreases the material amount towards the edges. Rather, it is the lower growth temperature causing the redshift. We stress that at a lower InAs deposition temperature, even less deposited indium should also be sufficient to reach the O-band and wavelengths beyond. All values can also be found in Table A2.

**Figure A3:**
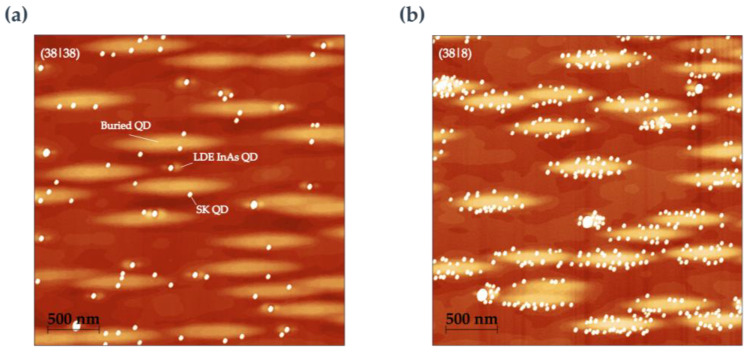
Surface morphology of sample $\#F_{0.60/GaAs/rot}^{0.4/Al1/rot}$ (#15744) at the center (38|38) and at position (38|8): (**a**) The color scale ranges from 0.4 nm (dark) to 6 nm (bright). The buried QDs and the LDE QDs have the same height as described in Figure A2. The SK QDs have a height of ~13 nm. (**b**) The color ranges from 0.3 nm (dark) to 10 nm (bright). The small bright dots have a height of ~12 nm and the large dots up to 35 nm.

Figure 9e illustrates the density of the buried QDs (identified by surface hillocks) and the surface LDE QDs. Representative AFM images are shown in Figure A3. In addition to the buried LDE QDs, buried SK QDs can also contribute to the density of the overall buried QDs. A decrease in density of the surface LDE QDs towards the edge indicates a similar behavior of the buried LDE QDs. This would lead to the conclusion that SK QDs are increasingly formed towards the edge, which is consistent with previous observations.

In contrast to the GaAs QDs, the proportion of possible tunable LDE InAs QDs is significantly lower (see Figure 9f). One reason for this is the larger inhomogeneous broadening and resulting laser power broadened FWHM. Nevertheless, with a ±12.5 meV tuning range, 40% of the QDs can still be tuned to a wavelength even with radii greater than 30 mm. For smaller ranges, the homogenization would have to be optimized.

## 5. Discussion

### 5.1. Gradient Deposition of the QD Filling Material

In contrast to the SK-method where large density variations and only slight wavelength variation result from gradient deposition growth, in LDE-related QD nucleation the density is solely determined by the Ga or Al droplet density. It does not show a strong dependence on the amount of etch material if a critical amount is exceeded. However, we find that, in both LDE QD variants, the QD material quantity strongly impacts the QD emission wavelength, with stable regions of only small material amount related wavelength shifts.

For the LDE GaAs QDs, there is a continuous shift (at constant Al-etching material) due to the different hole filling, which flattens out with larger filling quantities. This could be due to the fact that at a certain point the holes are filled to the maximum and the nanohole is no longer an attractor for adatoms. Since the effective etching material amount influences the hole depth, this also has an influence on the emission wavelength and explains the different emission wavelengths of samples $\#D_{0.60/GaAs/rot}^{0.3/Al2/rot}$–$\#F_{0.60/GaAs/rot}^{0.4/Al1/rot}$ despite the same GaAs filling quantity.

For the LDE InAs QDs, we again find that the hole density determines the QD density and is a robust parameter against flux and temperature inhomogeneities. The dependence of the wavelength on the filling material and temperature, however, can be significantly stronger. Here, the holes form nucleation centers for the InAs QDs and thus also enable the formation of QDs at a deposition quantity that would not be sufficient for flat substrate SK QD nucleation. Thus, the material amount gives rise to a strong redshift with a sort of plateau that is relatively robust to flux inhomogeneities. The impact from the temperature distribution can be strong. As the amount of indium increases, the QDs become larger and shift into the red spectral range. This increase is steep and suddenly flattens out sharply (transition from area A to B) and is limited to a maximum emission wavelength of 1350 nm. The exact reason for this behavior is still unclear. However, it is clear that the QD density is fixed by the nanoholes, and “unnatural” growth happens with a steep increase in QD size with the material amount. This growth must be limited at some point. Quantum dot size limits could arise, e.g., from strain energy minimization, geometric constraints, (strain-induced) erosion during overgrowth, and dislocation defects in oversized dots or their matrix, often acting in combination. Further investigations are in progress. As soon as the threshold for the critical amount of InAs for SK QDs is exceeded at the transition from area B to C, we see a drop in intensity, clearly indicating that a dislocation model quenching emission needs to be considered. For C, we observe a clear change in the spectrum. We assume that only the standard SK QDs are still emitting here, which in turn are slightly blue-shifted. The QDs in the filled holes are no longer optically active, according to our interpretation. The higher PL intensity therefore results from the higher SK QD density. The amount of Ga-etching material does not appear to have any influence on the emission wavelength at the selected growth parameters [55].

### 5.2. Influence of Different Parameters on Homogeneity

The growth parameters needed for a specific design wavelength can be determined from one or, if needed, a few gradient samples grown within the same growth campaign. The parameters are the required effective etching amount and the GaAs or InAs material filling amount from regions that are robust towards variations in material flux, i.e., overfilled holes in the case of GaAs QDs or region B in the case of LDE InAs QDs.

Further steps to enable homogeneous growth over a large area are a uniform material deposition, enabled by the shutter-synchronous rotation, and a homogeneous temperature.

In the case of the InAs QDs, tiny disturbances such as not perfectly matched synchronization and especially temperature variations impacted the homogeneity. The indium cell profile (see Figure A1), and thus the material deposition, decrease sharply towards the edges. However, observation of a redshift and larger level splitting shows that the amount of material increases as the temperature also decreases towards the edges. This means that the temperature effect has a major impact. High temperature enables surface indium material desorption and strong diffusion. A lower temperature at the wafer edge allows for higher QDs and/or higher InAs content in the QDs. This indicates that growth with material amounts corresponding to region B combined with a homogeneous temperature profile is a route to homogeneous O-band QD fabrication. To counteract this effect, a more homogeneous sample heater design or a heat reflector ring as a wafer platen could be used.

LDE GaAs QDs, on the other hand, show a broad homogeneous profile demonstrating the robustness of the method for these QDs even under uneven temperature and Al-flux conditions. This facilitates a large wafer area for SPS fabrication with ideal QD density, QD homogeneity, and QD wavelength within electrical tuning range. The wafer profile is still slightly influenced by the Ga effusion cell profile (see Figure A1). However, as we chose the Ga amount to be high enough to fill the nanoholes completely, the impact is small and only visible for the outer region. As with the LDE InAs QDs, the cell profile of the etching material cell is less important, provided that sufficient material is deposited, and one is far enough away from a transition.

The nearly identical emission wavelength profiles of samples #E and #F demonstrate that the shutter-synchronous deposition method achieves high repeatability. Furthermore, its scalability and adaptability are highlighted by its successful application to LDE InAs QDs. The repeatability of growth parameters is also evident in the exact placement of the transition in the center of samples #A to #C and the precise positioning of the O-band QD regions (see also [55]). This demonstrated reproducibility verifies the robustness and reliability of the method.

## 6. Conclusions

This study presents a rapid parameter optimization method to determine the ideal growth conditions for achieving a desired design wavelength. It demonstrates the potential of shutter-synchronized material deposition in MBE for fabricating QDs with unprecedented density and wavelength uniformity over a large area. These results pave the way for scalable production of QD wafers at rubidium memory and telecom wavelengths, which are critical for QD-based quantum communication technologies.

Key outcomes include achieving narrow wavelength spreads (<2 nm) for GaAs QDs over a large area (~*D* = 50 mm) and demonstrating the feasibility of O-band LDE InAs QDs with significant improvements in density and growth homogeneity. With a tuning range of ±12.5 meV and a wafer diameter of 50 mm, we find that 98% of all QDs can be brought into mutual resonance. Even with a small tuning range of only ±1 meV, at least 40% of all QDs at every point on the wafer within a diameter of 40 mm can be tuned into exact mutual resonance. At a typical area density of 1 QD/µm^2^, this corresponds to more than half a billion QDs on a single wafer.

Future work should focus on optimizing temperature distribution during growth to extend uniformity over larger areas and refining QD density and emission wavelength control. While GaAs QDs already exhibit excellent quantum-photonic behavior, further work is needed to demonstrate that O-band LDE-based QDs can achieve similar performance.

In conclusion, the advancements demonstrated in this study are crucial for the deployment of quantum-photonic devices at industrial scales, marking a significant step towards the scalable production of high-quality QD wafers.

## Figures and Tables

**Table 1:** Growth parameters of the LDE GaAs QDs. The superscript indicates the layer thickness equivalent etching quantity, the etching material, and the deposition method. The subscript contains the fill amount, the filling material, and also the deposition method for filling nanoholes. The five-digit number is the internal sample number.

Sample	QD Growth Mode	As-BEP (µTorr)	Al-Etch (nm)	Al Cell	GaAs Fill (nm)
$\#A_{1.00/GaAs/grad}^{0.3/Al2/grad}$/15379	Gradient	0.46	0.3	#2	1.00
$\#B_{0.60/GaAs/grad}^{0.3/Al2/grad}$/15384	Gradient	0.49	0.3	#2	0.60
$\#C_{0.44/GaAs/grad}^{0.3/Al2/grad}$/15389	Gradient	0.51	0.3	#2	0.44
$\#D_{0.60/GaAs/rot}^{0.3/Al2/rot}$/15530	Shutter sync.	0.34	0.3	#2	0.60
$\#E_{0.60/GaAs/rot}^{0.4/Al2/rot}$/15541	Shutter sync.	0.35	0.4	#2	0.60
$\#F_{0.60/GaAs/rot}^{0.4/Al1/rot}$/15544	Shutter sync.	0.27	0.4	#1	0.60

**Table 2:** Wavelength range of GaAs QD samples with gradient-deposited filling amount. Δλ corresponds to the span of the peak wavelength.

Sample	$\#A_{1.00/GaAs/grad}^{0.3/Al2/grad}$	$\#B_{0.60/GaAs/grad}^{0.3/Al2/grad}$	$\#C_{0.44/GaAs/grad}^{0.3/Al2/grad}$
GaAs filling (nm)	1	0.6	0.44
Wavelength range (nm)	805–770	785–740	765–710
Δλ (nm)	35	45	55

**Table 3:** Growth parameters of the LDE InAs QDs.

Sample	QD Growth Mode	Ga-Etch (nm)	InAs Cycles
$\#G_{12.25/InAs,grad}^{0.84/Ga/rot}$/15736	Gradient	0.84	12.25
$\#H_{12.25/InAs/rot}^{0.84/Ga/rot}$/15744	Shutter sync.	0.84	12.25

## Data Availability

The original data presented in this study are openly available at https://doi.org/10.6084/m9.figshare.27981563 (accessed on 16 January 2025).

## Appendix A

Table A1 and Table A2 show the results of the statistical analysis. The mean value and standard deviation are given for the emission wavelength and the FWHM. Δλ indicates the total peak wavelength variation. Proportion of QDs here means the proportion of ensemble peaks in a certain range around the mean wavelength.

**Table A1:** Results of the shutter-synchronized grown GaAs QDs.

Sample	$\#D_{0.60/GaAs/rot}^{0.3/Al2/rot}$			$\#E_{0.60/GaAs/rot}^{0.4/Al2/rot}$			$\#F_{0.60/GaAs/rot}^{0.4/Al1/rot}$		
Diameter (mm)	30	45	60	30	45	60	30	45	60
Emission wavelength (nm)	772.6 ± 0.9	771.3 ± 1.7	768 ± 5	788.4 ± 0.5	788.3 ± 0.6	787.5 ± 1.3	801.8 ± 0.1	802.3 ± 0.2	801.1 ± 1.0
Ensemble-PL_FWHM (meV)	32.7 ± 0.6	33.0 ± 0.9	35.0 ± 3.0	17.3 ± 0.3	17.3 ± 0.4	17.4 ± 0.5	12.86 ± 0.16	12.66 ± 0.27	12.6 ± 0.3
Δλ (nm)	4.4	7.5	21.1	2.0	2.7	5.7	0.6	1.2	4.1
Proportion of QDs (±5 nm)	100	100	66.1	100	100	100	100	100	100
Proportion of QDs (±2 nm)	97.4	74.1	24.7	100	100	89.8	100	100	93.3

**Table A2:** Results of the shutter-synchronized grown InAs QDs.

Sample	$\#H_{12.25/InAs/rot}^{0.84/Ga/rot}$			
Diameter (mm)	0	30	45	60
Emission wavelength (nm)	~1315	1315.3 ± 1.3	1317 ± 3	1323 ± 7
Ensemble-PL_FWHM (meV)	~30	31.3 ± 1.6	31.7 ± 1.9	30.6 ± 2.6
Δλ (nm)	-	8.0	14.4	28.9
Peak wavelength (±5 nm)	-	100	92.1	35.6
Proportion of QDs (±2 nm)	-	74.4	45.2	13.19

The growth of the surface QDs could not be supervised due to external circumstances, so a lower temperature or an indium-shutter malfunction could be the cause of a slightly increased InAs quantity. Nevertheless, the higher density of the SK QDs towards the edge confirms a wafer temperature-related increase in the effective amount of indium despite an indium profile that is contrary to this (see Figure A1).
